# Author Correction: Deep brain activities can be detected with magnetoencephalography

**DOI:** 10.1038/s41467-021-23215-8

**Published:** 2021-04-30

**Authors:** Francesca Pizzo, N. Roehri, S. Medina Villalon, A. Trébuchon, S. Chen, S. Lagarde, R. Carron, M. Gavaret, B. Giusiano, A. McGonigal, F. Bartolomei, J. M. Badier, C. G. Bénar

**Affiliations:** 1 Aix Marseille Univ, INSERM, INS, Inst Neurosci Syst, Marseille, 13005 France; 2 APHM, Timone Hospital, Epileptology and cerebral rhythmology, Marseille, 13005 France; 3 APHM, Timone Hospital, Functional and Stereotactic Neurosurgery, Marseille, 13005 France; 4 INSERM UMR894, Paris Descartes university, GHU Paris Psychiatrie Neurosciences, 75013 Paris, France

**Keywords:** Single-channel recording, Magnetoencephalography, Neurophysiology, Biomedical engineering

Correction to: *Nature Communications* 10.1038/s41467-019-08665-5, published online 27 February 2019.

The original version of this Article contained an error in the spelling of the author Francesca Pizzo which was incorrectly given as F. Pizzo. This has now been corrected in both the PDF and HTML versions of the Article.

