# Supplementary material for: Deep brain activities can be detected with magnetoencephalography
Source: Nat Commun. 2019 Feb 27;10:971. doi: 10.1038/s41467-019-08665-5 (PMC6393515; doi:10.1038/s41467-019-08665-5)

Supplementary information file

Deep brain activities can be detected with magnetoencephalography

Pizzo et al.

Supplementary Figure 1. Mesial ICA components. One example of a mesial ICA component per patient is reported in each line of the figure (for P5, P8 and P12 see Fig 1, 2 and 3). For each component is shown: SEEG-MEG ICA temporal correlation (on x axis: time (s) and on y axis: amplitude (a.u.)), ICA MEG topography and ICA source localization (color bar showing the map score for P1 (double dipole fit - see Methods for details) and GOF (a.u.) for all other components)

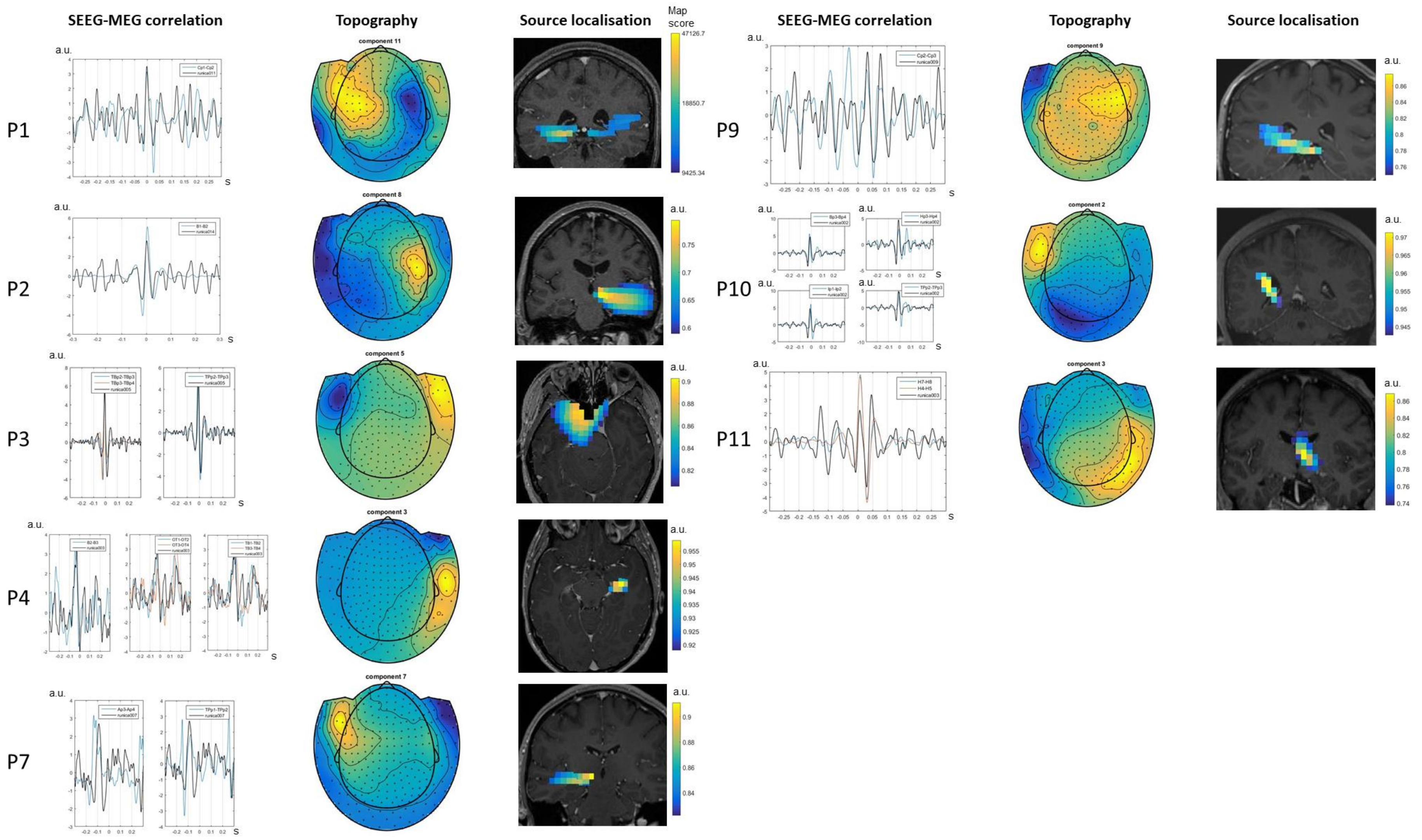

Supplement: Supplementary file 1 — Supplementary Information [file 41467_2019_8665_MOESM1_ESM.pdf]
