# Supplementary material for: Deep brain activities can be detected with magnetoencephalography
Source: Nat Commun. 2019 Feb 27;10:971. doi: 10.1038/s41467-019-08665-5 (PMC6393515; doi:10.1038/s41467-019-08665-5)
Supplement: Supplementary file 2 — Supplementary Data 1 [file 41467_2019_8665_MOESM2_ESM.pdf]

Supplementary data file

Deep brain activities can be detected with magnetoencephalography

Pizzo et al.

Supplementary Data 1. MEG ICA characteristics

Patient number	Marker position (structure)	Leralisation	number event marked	Filter	Continuous analysis	Network type	Visibility vs background	Visibility during event	SEEG-correLed structures	Subdivision Hipp	Neocortex	Single dipole GOF (double dipole GOF)	Source localization [mesial vs other]	Structure included in source localization CI
P1	hipp	Le	108	12-60Hz	no	M			Hipp	tale		0.72 (0.87)	M	Yes (Hipp; double dipole)
					yes	M			Hipp	tale		0.96	central-post central	NA
P2	hipp	R	185	12-60Hz	no	M			Hipp; PH	head		0.47	NA	NA
					yes	M			Hipp	head		0.8	M	Yes (Hipp)
					yes	M			Hipp; PH	head		0.75	NA	NA
P3	hipp	Le	183	2-60Hz	no	ML	20		Amy; PeriRh; NC		T			
						M	100		Hipp	tale		0.86	Le Insulo-opercular	NA
				12-60Hz	no	M	15		PeriRh; CS			0.9	M	Yes (PeriRh; CS)
						M	100		Hipp; PH	head; tale		0.43 (0.79)	NA	NA
				2-60Hz	yes	M	15		PeriRh			0.9	M	No (Hipp)
						M	150		Hipp; PeriRh	head		0.94	M	Yes (Hipp)
						L	30		NC		T	0.82	M, BiL T pole	NA
						M			PeriRh			0.36 (0.5)	NA	NA
				12-60Hz	yes	M	10		CS			0.85	M	No

														(thalamus)
						M			PeriRh			0.75<br>(0.95)	M	Yes (PeriRh, double dipole)
						M			CS			0.7	NA	NA
						M	50		Hipp	head		0.42<br>(0.8)	NA	NA
						M			Hipp; PH			0.73	NA	NA
P4	hipp	R	99	2-60Hz	no	ML	5		Hipp; CS; FusG; NC	head	T			
						L			NC		T	0.9	L	No (parietal)
						ML	50		Hipp; CS; FusG; NC	head	T			
						eL			PeriRh; PH; CS; Amy; OTS; TH					
				12-60Hz	no	ML			Hipp; CS; FusG; NC	head	T			
						ML	10		Hipp; CS; FusG; NC	head	T			
						ML			Hipp; EC; CS; FusG; NC	head	T			
				2-60Hz	yes	ML	5	15	Hipp; CS; FusG; NC	head	T			
						ML			CS; FusG; NC	head	T			
						M			Hipp	head		0.92	M	No (thalamo-cingular

														post)
						ML	30		Hipp; CS; FusG; NC	head	T			
						eL			Amy; PHG; CS; TH; OTS; NC		T			
				12-60Hz	yes	ML			Hipp; CS; FusG; NC	head	T			
						M			CS			0.87	Le central	NA
						L			NC		T	0.88	L	No (P)
						ML			CS; PHG; FusG; NC		T			
						eL			Hipp; PH; CS; NC; TH	head	T			
						ML			Hipp; CS; NC	head	T			
						ML	50		Hipp; CS; PH; FusG; NC	head	T			
	hipp	Le	45	2-60Hz	no	eL	5	30	Amy; Hipp; PH; PeriRh; NC; TH	head	T			
						M			Hipp; CS; PH; FusG			0.95	M	Yes (Hipp; CS; PH; FusG)
				12-60Hz	no	eL	10		Amy; NC; TH; Hipp ; PH; PeriRh	head				
						L			NC		O	0.88	L	Yes
						ML			Hipp; CS; FusG; OTS;	head	T			

									NC					
				2-60Hz	yes	ML	30		Hipp; CS; FusG; OTS; NC	head	T			
						M	10		Amy			0.7	NA	NA
						M			PeriRh			0.86	Central-post central	NA
						ML			Hipp; CS; NC	head	T			
						eL	5		Amy; NC; CS; PH; PeriRh		T			
				12-60Hz	yes	L	20		NC		T	0.91	L	No (P)
						eL	10		Hipp; NC; CS; TH; PeriRh	head	T			
						ML			Hipp; NC; CS	head	T			
P5	hipp	R	16	2-60Hz	no	ML	10		EC; NC		T			
						M	5	10	Amy			0.92	Me	Yes (amy)
P6	hipp	Le	18	12-60Hz	yes	M			CS			0.95	Le prefrontal	NA
P7	amy	R	33	2-60Hz	no	ML	30		Amy; OTS; PeriRh; NC		T			
				12-60Hz	no	ML			Amy; PeriRh; NC		T			
				2-60Hz	yes	ML			Amy; OTS; PeriRh; NC		T			
	hipp	Le	22	2-60Hz	no	M			Amy; PeriRh			0.92	M	Yes (Amy; PeriRh)
				12-60Hz	no	M			Hipp	head		0.79 (0.92)	M	Yes (Hipp, double

														dipole)
P8	hipp	R	23	2-60Hz	no	*m*eL	20		Hipp; TH	head		0.93	M	Yes (Hipp; TH)
				2-60Hz	no	eL	20		PeriRh; NC; TH		T			
				12-60Hz	no	*m*eL	15		Amy; Hipp; TH	head		0.82	M	Yes (Hipp; TH)
				12-60Hz	yes	*m*eL	15		Hipp; TH	head		0.84	M	Yes (Hipp; TH)
P9	hipp	Le	38	12-60Hz	no	M			Hipp	tale		0.88	M	Yes (Hipp)
P10	amy	Le	32	2-60Hz	no	ML	30		Amy; OTS					
						L			NC		T	0.65 (0.93)	L	No (P)
				12-60Hz	no	*m*eL	10		PeriRh; ins; Hipp; TH	head		0.97	M	Yes (Hipp; TH)
						ML	30		Hipp; NC	head	T			
						L			NC		P	0.39 (0.67)	NA	NA
				2-60Hz	yes	ML	15		OTS					
						L			NC		P; T	0.78	L	Yes (P)
				12-60Hz	yes	*m*eL			PeriRh; TH; Hipp; Amy; ins	head		0.9	M	Yes (TH, Hipp, Ins)
						L			NC		T	0.86	L	No (P)
P11	hipp	R	55	12-60Hz	no	*m*eL	50		TH; putamen; ins			0.83	M	Yes (TH)
				2-60Hz	yes	*m*eL			TH			0.87	M	Yes (TH)
				12-60Hz	yes	*m*eL			TH; FusG			0.88	M	Yes (TH)
P12	hipp	Le	53	2-60Hz	no	*m*eL	50		Hipp; TH	head		0.9	M	Yes

														(Hipp; TH)
						ML	15		Amy; NC		T			
				12-60Hz	no	M	10	50	Amy; Hipp	head		0.96	M	No (thal)
						*m*eL	50		TH			0.89	M	Yes (TH)
				2-60Hz	yes	M	5		Amy			0.92	M	No (Hipp)
				12-60Hz	yes	M	20		Hipp; Amy			0.86	M	Yes (Hipp)
						M	5		Hipp; Amy			0.92	Le occipital	NA
						eL			Hipp; NC	tale	T; OF			
						M			Amy			0.68 (0.91)	M	No (Hipp: double dipole)
P13	amy	Le	145	2-60Hz	no	ML	10	30	Amy; EC; OTS					
						eL			Ins; NC; EC		T			
						L			NC		T	0.79 (0.95)	L	Yes (T)
						L			NC		T	0.89	L	Yes (T)
						eL			Amy; OTS; Ins; NC		T			
				12-60Hz	no	L			NC		F	0.97	L	No (P)
						L			NC		F	0.91	L	Yes (F)
						ML	20		Amy; OTS; NC		F; T			
						L			NC		T	0.89	L	Yes (T)
				2-60Hz	yes	ML			EC; NC		T			
						L	50		NC		T	0.82 (0.96)	M (Thalamic/T pole)	NA

						eL	15		Amy; OTS; Ins; NC		T			
						L			NC		T	0.78	L	Yes (T)
						eL			NC		T; OF			
				12-60Hz	yes	ML			Amy; OTS; NC		T; F			
P14	amy	R	86	2-60Hz	no	L			NC		T	0.93	L	Yes (T)
						L			NC		T	0.77 (0.91)	L	Yes (T)
				12-60Hz	no	L			NC		T	0.95	L	Yes (T)
						L			NC		T; F	0.73 (0.97)	L	Yes (T)
						L			NC		T; F	0.88	L	Yes (T)
						L			NC		T; F	0.83	L	Yes (T)
						ML			Amy; NC		T; F			
						L			NC		T	0.88	L	Yes (T)
				2-60Hz	yes	L			NC		T	0.91 (0.98)	L	Yes (T)
						L			NC		T	0.94	L	Yes (T)
						L			NC		T	0.6 (0.82)	L	No (P)
				12-60Hz	yes	L			NC		T	0.93	L	Yes (T)
						L			NC		T	0.91	L	Yes (T)
						L			NC		T	0.93	L	Yes (T)
						L			NC		T; F	0.95	L	Yes (T)
						L			NC		T	0.96	L	Yes (T)

Legenda: hipp- hippocampus-; amy – amygdala-; R –right-; Le –left-; M – mesial-; L – Lateral-; ML –mesioLateral-; eL – extended limbic-; PH – parahippocampal gyrus-; PeriRh –preirhinal cortex-; CS –collateral sulcus- FusG – fusiform gyrus-; OTS – occipito-temporal sulcus-; TH –thalamus-; EC entorhinal cortex-; ins – insula-; NC – neocortex-; OF – orbitofrontal ; T – temporal-; F –frontal-; P –parietal- O -occipital. NA: not applicable
