# Supplementary material for: Deep brain activities can be detected with magnetoencephalography
Source: Nat Commun. 2019 Feb 27;10:971. doi: 10.1038/s41467-019-08665-5 (PMC6393515; doi:10.1038/s41467-019-08665-5)
Supplement: Supplementary file 4 — Reporting Summary [file 41467_2019_8665_MOESM4_ESM.pdf]

nature research

Corresponding author(s): Francesca Pizzo, Christian Benar

Last updated by author(s): 2018 12 20

# Reporting Summary

Nature Research wishes to improve the reproducibility of the work that we publish. This form provides structure for consistency and transparency in reporting. For further information on Nature Research policies, see Authors & Referees and the Editorial Policy Checklist.

## Statistics

For all statistical analyses, confirm that the following items are present in the figure legend, table legend, main text, or Methods section.

| n/a | Confirmed | |
|---|---|---|
| ☐ | ☒ | The exact sample size ($n$) for each experimental group/condition, given as a discrete number and unit of measurement |
| ☐ | ☒ | A statement on whether measurements were taken from distinct samples or whether the same sample was measured repeatedly |
| ☐ | ☒ | The statistical test(s) used AND whether they are one- or two-sided<br>*Only common tests should be described solely by name; describe more complex techniques in the Methods section.* |
| ☐ | ☒ | A description of all covariates tested |
| ☐ | ☒ | A description of any assumptions or corrections, such as tests of normality and adjustment for multiple comparisons |
| ☐ | ☒ | A full description of the statistical parameters including central tendency (e.g. means) or other basic estimates (e.g. regression coefficient) AND variation (e.g. standard deviation) or associated estimates of uncertainty (e.g. confidence intervals) |
| ☐ | ☒ | For null hypothesis testing, the test statistic (e.g. $F$, $t$, $r$) with confidence intervals, effect sizes, degrees of freedom and $P$ value noted<br>*Give P values as exact values whenever suitable.* |
| ☒ | ☐ | For Bayesian analysis, information on the choice of priors and Markov chain Monte Carlo settings |
| ☒ | ☐ | For hierarchical and complex designs, identification of the appropriate level for tests and full reporting of outcomes |
| ☐ | ☒ | Estimates of effect sizes (e.g. Cohen's $d$, Pearson's $r$), indicating how they were calculated |

*Our web collection on statistics for biologists contains articles on many of the points above.*

## Software and code

Policy information about availability of computer code

| | |
|---|---|
| Data collection | Brainproducts (Brain Vision recorder), 4D Neuroimaging |
| Data analysis | MATLAB R2015B |

For manuscripts utilizing custom algorithms or software that are central to the research but not yet described in published literature, software must be made available to editors/reviewers. We strongly encourage code deposition in a community repository (e.g. GitHub). See the Nature Research guidelines for submitting code & software for further information.

## Data

Policy information about availability of data

All manuscripts must include a data availability statement. This statement should provide the following information, where applicable:

- Accession codes, unique identifiers, or web links for publicly available datasets
- A list of figures that have associated raw data
- A description of any restrictions on data availability

Code availability.
The code used to generate the results that are reported in this study is available from the corresponding authors upon reasonable request

Data availability
All data supporting the findings of this study are available from the corresponding authors upon reasonable request.

# Field-specific reporting

Please select the one below that is the best fit for your research. If you are not sure, read the appropriate sections before making your selection.

☒ Life sciences ☐ Behavioural & social sciences ☐ Ecological, evolutionary & environmental sciences

For a reference copy of the document with all sections, see nature.com/documents/nr-reporting-summary-flat.pdf

# Life sciences study design

All studies must disclose on these points even when the disclosure is negative.

| | |
|---|---|
| Sample size | The only criterion for patient selection was the presence of at least one electrode located in the hippocampus head or in the amygdala showing interictal epileptic activity ("interictal spikes") during underwent a simultaneous SEEG MEG recording study. Fourteen patients were studied (8 female). |
| Data exclusions | No electrode located in the hippocampus head or in the amygdala showing interictal epileptic activity |
| Replication | Replicability was tested across patients in these very rare datasets |
| Randomization | not applicable |
| Blinding | This is a retrospective study on simultaneous MEG-SEEG recordings; blinding is not relevant |

# Reporting for specific materials, systems and methods

We require information from authors about some types of materials, experimental systems and methods used in many studies. Here, indicate whether each material, system or method listed is relevant to your study. If you are not sure if a list item applies to your research, read the appropriate section before selecting a response.

## Materials & experimental systems

| n/a | Involved in the study | |
|---|---|---|
| ☒ | ☐ | Antibodies |
| ☒ | ☐ | Eukaryotic cell lines |
| ☒ | ☐ | Palaeontology |
| ☒ | ☐ | Animals and other organisms |
| ☐ | ☒ | Human research participants |
| ☐ | ☒ | Clinical data |

## Methods

| n/a | Involved in the study | |
|---|---|---|
| ☒ | ☐ | ChIP-seq |
| ☒ | ☐ | Flow cytometry |
| ☒ | ☐ | MRI-based neuroimaging |

## Human research participants

Policy information about studies involving human research participants

| | |
|---|---|
| Population characteristics | Fourteen patients affected by pharmacoresistant epilepsy (8 female) . Patients' mean age at recordings was 31.5 years, mean age at epilepsy onset was 18.2 years and mean epilepsy duration was 15 years. |
| Recruitment | Patients who underwent a simultaneous intracerebral stereotaxic EEG (SEEG) - MEG recordings in the setting of pre-surgical evaluation of focal drug-resistant epilepsies at the Clinical Neurophysiology and Epileptology Unit, APHM, Marseille, France. |
| Ethics oversight | Comité de Protection des Personnes, Sud-Méditerranée I, ID-RCB 2012-A00644-39 |

Note that full information on the approval of the study protocol must also be provided in the manuscript.

## Clinical data

Policy information about clinical studies

All manuscripts should comply with the ICMJE guidelines for publication of clinical research and a completed CONSORT checklist must be included with all submissions.

| | |
|---|---|
| Clinical trial registration | NCT02875964 |
| Study protocol | https://clinicaltrials.gov/ct2/show/NCT02875964 |
| Data collection | october2012-present |

Outcomes

Primary objective: quantifying detectability of epileptic discharges; secondary objective: optimise signal processing methods
